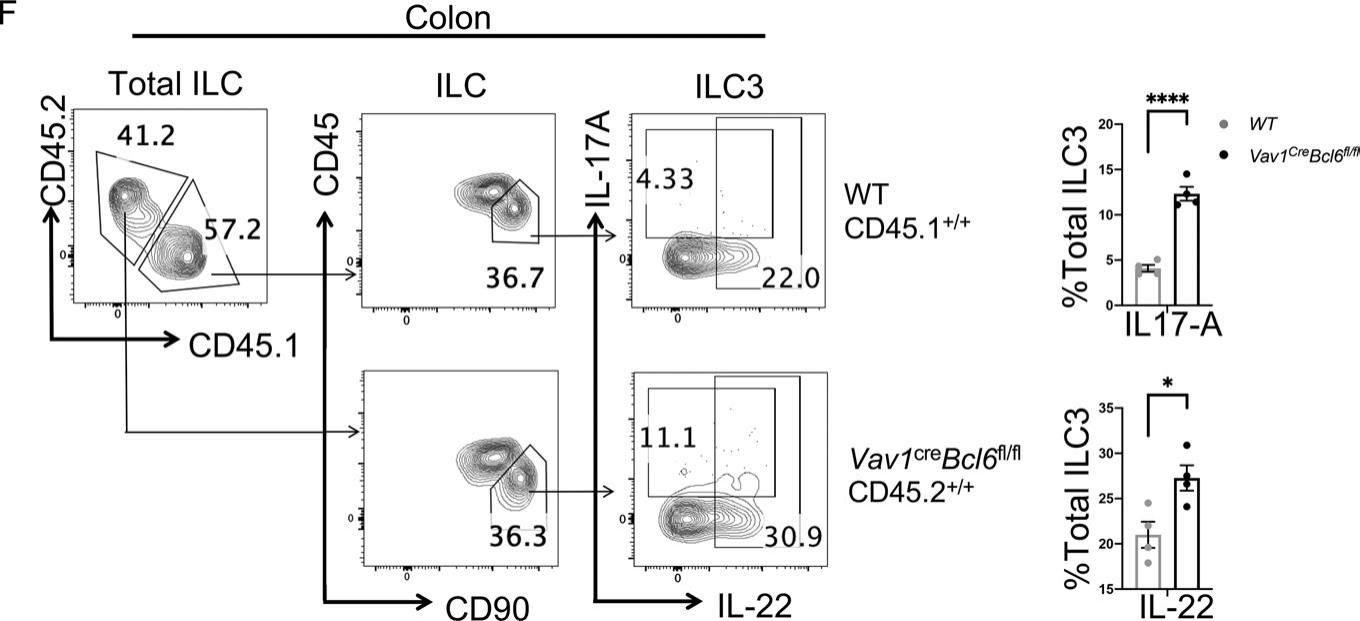# Correction: Cell autonomous expression of BCL6 is required to maintain lineage identity of mouse CCR6^+^ ILC3s

**DOI:** 10.1084/jem.2022044006302023c

**Published:** 2023-07-17

**Authors:** Yuling Li, Jing Ge, Xiaohong Zhao, Miao Xu, Mengting Gou, Bowen Xie, Jinling Huang, Qinli Sun, Lin Sun, Xue Bai, Sangnee Tan, Xiaohu Wang, Chen Dong

Vol. 220, No. 4 | https://doi.org/10.1084/jem.20220440 | January 18, 2023

The authors regret that in the originally published Fig. S1 F, two flow images representing CD45.1^+^ and CD45.2^+^ ILC3 populations in total ILCs were inadvertently duplicated. The corrected Fig. S1 F is shown here. This correction does not change the description, interpretation, or the original conclusions of the manuscript, and the legend remains unchanged. The error appears in PDFs downloaded before July 10, 2023.

**Figure:**